# Improved Benefit of SPECT/CT Compared to SPECT Alone for the Accurate Localization of Endocrine and Neuroendocrine Tumors

**DOI:** 10.4274/Mirt.80299

**Published:** 2012-12-20

**Authors:** Gonca G. Bural, Ashok Muthukrishnan, Matthew J Oborski, James M. Mountz

**Affiliations:** 1 University of Pittsburgh Medical Center, Department of Radiology, Pittsburgh, PA, USA

**Keywords:** tomography, emission-computed, single-photon, endocrine gland neoplasms, neuroendocrine tumors

## Abstract

**Objective:** To assess the clinical utility of SPECT/ CT in subjects with endocrine and neuroendocrine tumors compared to SPECT alone.

**Material and Methods:** 48 subjects (31 women;17 men; mean age 54±11) with clinical suspicion or diagnosis of endocrine and neuroendocrine tumor had 50 SPECT/CT scans (32 Tc-99m MIBI, 5 post treatment I-131, 8 In-111 Pentetreotide, and 5 I-123 MIBG). SPECT alone findings were compared to SPECT/CT and to pathology or radiological follow up.

**Results:** From the 32 Tc-99m MIBI scans, SPECT accurately localized the lesion in 22 positive subjects while SPECT/CT did in 31 subjects. Parathyroid lesions not seen on SPECT alone were smaller than 10 mm. In five post treatment I-131 scans, SPECT alone neither characterized, nor localized any lesions accurately. SPECT/CT revealed 3 benign etiologies, a metastatic lymph node, and one equivocal lesion. In 8 In-111 Pentetreotide scans, SPECT alone could not localize primary or metastatic lesions in 6 subjects all of which were localized with SPECT/CT. In five I-123 MIBG scans, SPECT alone could not detect a 1.1 cm adrenal lesion or correctly characterize normal physiologic adrenal uptake in consecutive scans of the same patient with prior history of adrenelectomy, all of which were correctly localized and characterized with SPECT/CT.

**Conclusion:** SPECT/CT is superior to SPECT alone in the assessment of endocrine and neuroendocrine tumors. It is better in lesion localization and lesion characterization leading to a decrease in the number of equivocal findings. SPECT/CT should be included in the clinical work up of all patients with diagnosis or suspicion of endocrine and neuroendocrine tumors.

**Conflict of interest:**None declared.

## INTRODUCTION

It has been a decade now since tomographic hybrid scanners including SPECT/CT (Single-photon emission computed tomography-computerized tomography) and PET/CT (positron emission tomography-computed tomography) have been introduced into nuclear medicine. These techniques provide a higher diagnostic accuracy than conventional non-tomographic scans (1).There has been a considerable emphasis on the benefits of PET/CT in oncology (2,3,4,5,6,7), but relatively less research proven emphasis on the SPECT/CT (8). Due to high investment expense and ongoing benefit to cost analysis, the clinical utility of SPECT/CT still warrants proof of data expressing its superiority. Incorporation of anatomic CT imaging with SPECT can increase the diagnostic capacity of these imaging modalities, predominantly in subjects presenting with the diagnosis or suspicion of endocrine and neuroendocrine tumors.Our aim was to assess the clinical utility of SPECT/CT compared with SPECT alone, in the assessment of subjects with endocrine and neuroendocrine tumors.

## MATERIALS AND METHODS

48 subjects (31 women; 17 men; mean age 54±11; age range 15-80 years) with clinical suspicion or diagnosis of endocrine and neuroendocrine tumors had 50 SPECT/CT scans performed on the dual head Siemens Symbia T6 SPECT/CT scanner between January 2010 and March 2012. Thirty-two of these scans were Tc 99m MIBI, 5 were post treatment I-131 scans, 8 were In-111 Pentetreotide scans, and 5 were I-123 MIBG scans.

**SPECT/CT Protocols**

All SPECT scans were performed using a 128x128 matrix, 30 seconds /stop, total of 64 stops obtaining 128 projections, using non circular orbit. All SPECT data was processed using filtered back projection, frequency cut off 0.5, order 5, and a second reconstruction using OSEM, 8 iterations and 16 subsets.

Tc-99m Sestamibi (MIBI) SPECT/CT scan: After intravenous administration of 1110 MBq Tc-99m sestamibi, immediate SPECT/CT images were obtained from the lower face to mid chest using a low energy collimator, energy setting at 140 KeV with 20% window. Following a 2-hour delay, repeat SPECT/CT images were obtained of the same level. MIP (maximum intensity projection) reconstructions were performed and the early and late images were compared side-by-side for distribution and retention of radiotracer.

I-131 Post treatment scan with SPECT/CT scan: One week after oral administration of therapeutic dose of I-131 raging between 1776–6031 MBq, anterior and posterior whole body planar images were obtained using a high energy collimator, energy settings at 364 KeV with 20% window and camera speed of 8 cm/min. SPECT/CT images of the neck as well as the regions with equivocal findings were obtained.

In-111 pentetreotide scan with SPECT/CT scan: 4 hours after the intravenous injection of the 222 MBq In-111 pentetreotide, whole body anterior and posterior planar images were obtained using a medium energy collimator, energy settings at 172 KeV and 247 KeV with 20% window and 10cm/min. Approximately 24 hours later, repeat whole body images in the anterior and posterior views, as well as SPECT/CT images of the regions with equivocal findings were obtained.

I-123 MIBG (Metaiodobenzylguanidine) scan with SPECT/CT scan: 24 hours after the intravenous injection of 400 MBq I-123 MIBG, whole body anterior and posterior images were obtained using a low energy high resolution collimator, energy settings at 159 KeV with 20% window, and 9cm/min. In addition SPECT/CT images of the regions with equivocal findings were obtained.

CT scan: After oral administration of 20 cc gastroview mixed with 900 cc clear non-carbonated liquid over the course of an hour, axial CT images of the area of interest were obtained using 2.5 mm collimation, 130 kVp, mAs of 20-60 for neck, and mAs of 40-80 for chest, abdomen, pelvis and extremities, depending on body habitus, and pitch of 0.8 with a Siemens Symbia T6 SPECT/CT scanner.

**Subjects**

32 subjects with elevated parathyroid hormone levels had 32 MIBI scans, all of which were before a parathyroid surgery. In these subjects lesion detection and localization with SPECT alone was compared to SPECT/CT. Findings were correlated with pathology. For the other scans, including five post treatment I-131 scans in 5 subjects with papillary thyroid cancer, eight In-111 Pentetreotide scans (the primary tumor was well differentiated pancreatic endocrine neoplasm in 4 subjects, well differentiated neuroendocrine neoplasm of small bowel in one subject, carcinoid tumor of the small bowel in one subject, gastrinoma in one subject, and gastric carcinoid tumor in one subject), and five I-123 MIBG scans in 4 subjects with suspicion of primary or recurrent pheochromocytoma, lesion detection, characterization, and localization were compared between planar and SPECT and planar and SPECT/CT images. The planar images and the SPECT alone component of the studies were interpreted and the findings were recorded. Planar and SPECT/CT images were then evaluated and the findings were recorded. Planar image findings and SPECT findings were compared to planar and SPECT/CT findings. All findings were correlated with histopathology when available, or radiologic follow-up. 

## RESULTS

48 subjects with clinical suspicion or diagnosis of endocrine and neuroendocrine tumors had 50 SPECT/CT scans. Localization or characterization of scan findings with SPECT alone and with SPECT/CT is summarized in Table 1.

In 32 subjects who had a MIBI scan, SPECT correctly identified and localized the hyper functioning parathyroid tissues in 22 subjects (61%), while SPECT/CT correctly identified and localized the hyper functioning parathyroid tissues in 31 subjects (97%). Histopathology revealed adenoma in 29 subjects, 3 hyperplastic glands in 3 subjects. Parathyroid lesions not detected with SPECT alone in 10 subjects all had size less than 10 mm.

**Figure 1:**
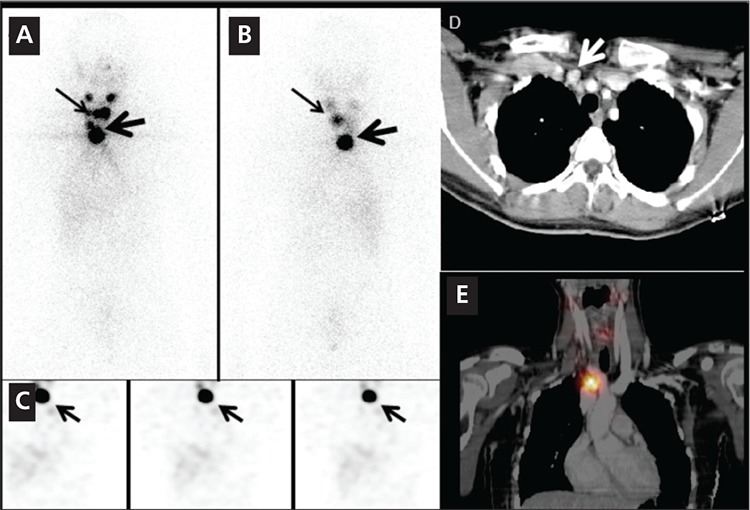
A 15 year-old girl with papillary thyroid cancer and totalthyroidectomy underwent radioiodine therapy with 3700 MBq I-131. The1 week post-therapy anterior (A) and posterior (B) whole body imagesshowed bilateral intense tracer uptake in the region of the submandibularglands and focal intense uptake in the thyroid resection bed (thin arrows).A suspicious focus of intense increased tracer uptake is noted in the lowerneck/upper mediastinum, (thick arrows, A,B). Selected coronal SPECTimages (C) of the neck obtained on the same day show focal intense uptakein the upper mediastinum (arrows, C). SPECT alone does not localize theuptake. Axial CT image (D) shows an enlarged 1.3x1.2 cm right upperparamediastinal lymph node (white arrow). Coronal fused image (E) showsabnormal increased tracer uptake in the enlarged lymph node seen on CT.This lymph node was correctly characterized and reported as metastaticwith SPECT/CT findings. Surgical resection of the lymph node revealedmetastatic papillary thyroid cancer

**Figure 2:**
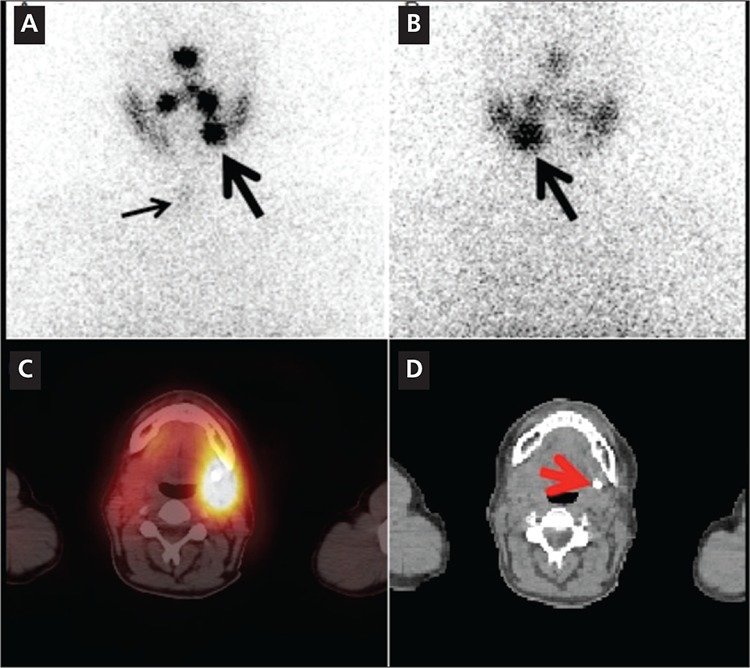
A 42 year-old woman with papillary thyroid cancer and totalthyroidectomy underwent radioiodine therapy with 6031 MBq I-131. 1week post-therapy anterior (A) and posterior (B) neck/chest images showedmild uptake in the thyroid bed (small arrow, image A) consistent withminimal residual thyroid tissue. A focus of intense increased asymmetricuptake noted in the left inferior head/neck region was concerning for nodalmetastasis (large arrows, images A, B). Axial fused SPECT/CT and axial CTimages (C, D). Focus of asymmetric increased uptake localizes to thesubmandibular gland. CT images show an 8 mm calcified stone immediatelyinferior to the left submandibular gland, causing duct obstruction andasymmetric uptake in the salivary gland (red arrow, D). On SPECT alone thiswas suspected to be a metastasis, but the addition of CT provided accuratecharacterization as a benign finding

In 5 post treatment I-131 scans, SPECT alone neither characterized nor localized any malignant lesions or benign findings accurately. However, SPECT/CT correctly localized and/or characterized 3 benign etiologies in 3 different subjects, a malignant lesion in the 4th subject and an equivocal finding in the fifth subject. Figure 1 shows improved localization for a metastatic superior mediastinal lymph node. Figure 2 illustrates characterization advantage of SPECT/CT by showing an incidental submandibular gland duct stone causing obstruction which initially, on SPECT alone, was suspected to be a metastasis.

**Figure 3:**
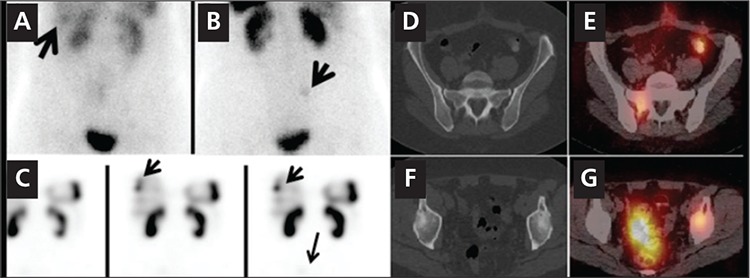
A 58 year-old woman with carcinoid tumor of the small bowel withknown liver metastases had an In-111 Pentetreotide scan. Anterior (A) andposterior (B) planar images of the abdominopelvic region show abnormal focaluptake is noted in the inferior right lobe of the liver (arrow, image A) and afaint focus of increased uptake is noted in the mid upper right hemi pelvis(arrow, image B). Selected coronal SPECT (C) images show foci of increaseduptake in the liver, corresponding to the known sites of liver metastases (smallarrows). A very faint focus of tracer uptake slightly above the bladder on theright is suspicious for a metastasis (arrow, C). However accurate interpretationof this finding is not possible with SPECT scan only. Axial SPECT/CT images atthe level of pelvis (D, E, F, G) show increased tracer uptake in the right sacrum(D,E), consistent with early bone metastasis, which correlated to the suspiciousfinding on planar posterior image (B). Another focus of abnormal increasedtracer uptake was noted in left acetabulum with predominantly scleroticchanges in the acetabulum (F, G). This lesion was not visible on planar imagesand was not localizable on SPECT alone. SPECT /CT however detected andlocalized this metastatic bone lesion

**Figure 4:**
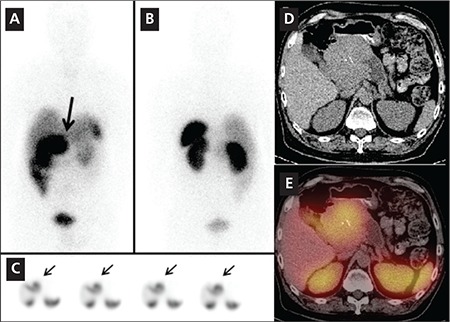
A 50 year-old man had a hyper vascular pancreatic head massdetected on an abdominal CT scan. The patient had an In-111 Pentetreotidescan to evaluate for tumor and to evaluate for distant metastases. Anterior(A) and posterior (B) whole body planar images from an In-111Pentetreotide scan showed a focus of intense abnormal tracer uptake in themid upper abdomen seen on anterior whole body image (arrow, A). Serialaxial SPECT slices (C) at the level of the abnormal tracer uptake (arrows)could not accurately localize the activity. The location could be falselyinterpreted as within the liver, small or large bowel or could be a metastaticupper abdominal mass. Axial CT scan at the level of the left adrenal glandshows a large, centrally calcified pancreatic head mass (D). Fused axial CTimage of SPECT/CT (E) at the same level shows increased tracer uptake inthe pancreatic head mass

In eight In-111 Pentetreotide scans, SPECT/CT was better than SPECT alone for lesion localization and characterization in the liver, pancreatic head, bones or lymph nodes. Pathologic confirmation was available for the 10 lesions (9 were malignant lesions one was benign), including liver metastases in 4 subjects, primary well differentiated endocrine tumor of the pancreas in one subject, retroperitoneal and superior mesenteric lymph node metastases in one subject and mediastinal and perirenal lymph node metastases in one subject and retroperitoneal lymph node in one subject. Two bone lesions did not have pathologic confirmation but, follow-up diagnostic CT scans confirmed the osseous metastases. Figures 3 illustrates detection and localization advantage for two osseous metastases. Figure 4 shows improved localization leading to better characterization of a 9 cm primary pancreatic head mass.

Four subjects had five I-123 MIBG scans as summarized in Table 1. In two subjects, large adrenal masses with intense I-123 MIBG uptake were visible on both planar whole body and SPECT scans. SPECT/CT did not reveal any additional abnormal findings. Surgical pathology was pheochromocytoma in both. In the third subject with suspicion of pheochromocytoma, a 1.1 cm left adrenal lesion had very mild asymmetric increased uptake, not very well identified on SPECT alone but better visualized on SPECT/CT images. The fourth subject with prior history of adrenelectomy, had normal physiologic adrenal uptake in consecutive scans. SPECT/CT correctly localized the uptake to the single hyperplasic adrenal gland and subsequently characterized this finding as benign normal finding.

## DISCUSSION

Computerized tomography (CT) is a conventional high-resolution anatomical imaging modality that excels at providing details on lesion location, size, and morphology. However this modality does not provide information regarding tumor physiology (9). Despite its high sensitivity and specificity, SPECT alone is substantially limited by low spatial resolution and its inability to provide anatomical detail. Dual-modality imaging systems which allow both functional and structural imaging to be performed during a single imaging session improve image quality in comparison to functional imaging only (10). Fusion imaging resulting from hybrid devices has been reported to be clearly giving better localization of disease and differentiation between physiologic and pathologic uptake (11,12,13,14). This study emphasizes the clinical utility of SPECT/CT over SPECT alone in the assessment of endocrine and neuroendocrine tumors.

In our study, the correct detection and localization of hyper functioning parathyroid lesions by SPECT/ CT (31 of 32 subjects) was superior to SPECT (22 of 32 subjects) alone. All lesions which were not detected on SPECT alone in 10 subjects were smaller than 10 mm. SPECT/CT has a clear superiority over SPECT in the detection and localization of parathyroid lesions smaller than 10 mm. This is most likely secondary to poor spatial localization ability of SPECT alone in the absence of a precisely registered CT, leading to its inability to confidently detect small sized lesions. One out of 32 subjects who had an 8 mm adenoma in the left parathyroid gland detected in surgery was incorrectly localized by SPECT/CT to the right. The suspicious uptake in the right was most likely due to statistical noise. In this subject, 8 mm left parathyroid gland lesion could have been missed due to small size of the lesion or tumor characteristics.

Our patient based sensitivity for dual phase MIBI and SPECT/CT was 97% and for SPECT alone 61% respectively. In a retrospective study using dual phase MIBI and SPECT/CT in 94 patients the sensitivity of SPECT/CT was reported as 92%, which is similar to our findings (15). In another study with 116 patients, the sensitivity for SPECT/CT was slightly lower than our results with 88%, and the sensitivity for SPECT was similar to our findings with 59% (16).

In five subjects with post-therapy I-131 scans, SPECT/CT correctly characterized areas of suspicious uptake in three different subjects as physiologic or benign: physiologic thymic uptake, physiologic bowel uptake and a submandibular gland duct stone causing obstruction. In the fourth subject with post therapy I-131 scan, the suspicious uptake in the upper chest region localized to a right superior mediastinal lymph node which biopsy was proven to be metastatic thyroid tissue. In the last subject SPECT/CT localized the uptake to be within a small nodule in the mesentery of the upper abdomen. Follow-up surgical removal of this lesion was biopsy proven to be a benign epithelial cyst.

Previous studies in subjects who had post therapy I-131 scans and SPECT/CT reported similar findings to our results: including reduction or elimination in uncertain findings and better lymph node localization (17,18,19).

In 8 subjects who had In-111 Pentetreotide scans, SPECT/CT imaging improved lesion localization compared to planar and SPECT imaging in multiple metastatic lesions in various locations. Even for the very large lesions, SPECT alone was not helpful in lesion localization. A 9 cm pancreatic head mass had focal intense uptake in the mid upper abdomen on the whole body images. By SPECT alone, it was unclear if this was a large abdominal lymph node, an organ involvement or intense bowel uptake. SPECT/CT localized this to the pancreatic head, and showed no other evidence of abdominal disease. For the smaller lesions SPECT/CT was helpful both for detection and in almost all cases, characterization. Our findings are in keeping with the current literature, documenting the diagnostic value of SPECT/CT over planar and SPECT imaging in the assessment of neuroendocrine tumors with In-111 Pentetreotide (20,21,22). Despite the overall benefits of SPECT/CT with In-111 Pentetreotide scans in identifying and localizing disease in the majority of subjects, it was misinterpreted in one subject. This subject had a pelvic mesenteric lymph node with mild uptake, which was characterized as suspicious for residual disease or recurrence at the surgical site however, histopathology revealed chronic inflammation and fat necrosis.

In 4 subjects who had five I-123 MIBG scans, 2 had very large left adrenal masses identified equally well both on SPECT and SPECT/CT (one was 10 cm and other was 5 cm) consistent with pheochromocytoma. However SPECT/CT was additionally helpful in these cases, as it did not reveal any other metastatic foci elsewhere in these subjects. The third subject had a prior right adrenal pheochromocytoma resection but remained symptomatic. In this case two SPECT/CT scans provided convincing evidence of an anatomically normal but minimally hyperplastic left adrenal gland. The fourth subject had mildly elevated vanillymandelic ascite (VMA) in the urine but normal plasma metanephrine levels. The I-123 MIBG scan with SPECT/CT detected a 1.1 cm left adrenal lesion with only mildly increased tracer uptake. Given these equivocal findings, a MRI scan was performed, which was normal. Clinically, the patient was diagnosed as having subclinical pheochromocytoma, and did not undergo surgery. A case report, with similar clinical picture to our subject, where subclinical pheochromocytoma was diagnosed on histopathology was published by Minako et al (23). Another study reported that I-123 MIBG SPECT or CT scanning alone were equally good for locating adrenal medullary pheochromocytoma but the combination of MIBG SPECT and CT makes it possible to distinguish between functioning and nonfunctioning adenomas (24).

Currently one major obstacle preventing routine clinical use of SPECT/CT is the high cost of SPECT/CT machines (25). However, we believe that as data continues to prove their clinical utility, this technology will become more widely available and a larger number of patients will be able to benefit from combined SPECT/CT imaging. 

## CONCLUSION

In this study we show that functional SPECT and anatomic CT data obtained as a single study have shown improvements in diagnostic imaging capability by improving lesion conspicuity, reducing false positives, and clarifying indeterminate lesions by better localization compared to SPECT alone. SPECT/CT is a very valuable and effective imaging tool in the assessment of patients with endocrine and neuroendocrine malignancies and should be included in the clinical work-up of these patients rather than SPECT alone. 

## Figures and Tables

**Table 1:**
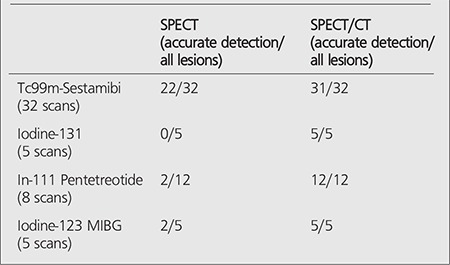
SPECT alone compared with SPECT/CT for accurate localizationand/or characterization of endocrine and neuroendocrine tumors
